# Cognitive Impairment in Bipolar Disorder and Schizophrenia: A Systematic Review

**DOI:** 10.3389/fpsyt.2013.00087

**Published:** 2013-08-08

**Authors:** Paul A. Vöhringer, Sergio A. Barroilhet, Andrea Amerio, Maria Laura Reale, Katherine Alvear, Derick Vergne, S. Nassir Ghaemi

**Affiliations:** ^1^Unidad de Trastornos del Ánimo, Clínica Psiquiátrica, Departamento de Psiquiatria, Facultad Medicina, Hospital Clínico Universidad de Chile, Santiago, Chile; ^2^Mood Disorders Program, Tufts Medical Center, Boston, MA, USA; ^3^Harvard School of Public Health, Boston, MA, USA; ^4^Escuela de Psicología, Universidad de los Andes, Santiago, Chile; ^5^Section of Psychiatry, Department of Neuroscience, University of Parma, Parma, Italy; ^6^Department of Psychiatry, Parmenio Piñero Hospital, Buenos Aires, Argentina; ^7^Universidad Diego Portales, Santiago, Chile; ^8^Tufts University School of Medicine, Boston, MA, USA; ^9^Director Mood Disorders Program, Tufts Medical Center, Boston, MA, USA

**Keywords:** systematic review, cognitive impairment, schizophrenia, bipolar disorder, working memory

## Abstract

**Aims:** Previous comparisons of cognitive decline among patients with bipolar disorder (BD) and schizophrenia (SZ) have found somehow quite similar profiles of deficits, but results have varied between studies. Therefore an extensive and thoughtful systematic review of the matter is warranted.

**Methods:** Studies were found through systematic search (PubMed) following PRISMA guidelines. To be included, studies must have assessed the following cognitive functions: executive functions, memory, IQ, attention-concentration, and perceptuomotor function. In order to make comparison between the two entities, studies should include BD patients with operationally defined euthymia, schizophrenic patients in remission, and third group of healthy control patients. Comparisons were made after controlling for years of schooling and residual affective symptoms.

**Results:** We found that overall both SZ and BD patients present deficits on all neurocognitive measures compared to healthy controls. In particular, SZ patients show more severe and pervasive cognitive deficits while BD patients present a milder and more confined impairment. In addition, evidence from the literature suggests that SZ and BD patients share a similar cognitive impairment profile with different degrees of deficits. Therefore, the difference between the two groups seems to be more quantitative (degree of deficit) rather than qualitative (profile), supporting a dimensional approach to the two clinical entities. Limitations of the present review includes the impossibility to control for effects of medication, varying time required for assessment across studies, illness diagnosis reliability, and course severity.

**Conclusion:** Patients with BD might exhibit a cognitive impairment that could be similar to SZ in terms of their profile, although patients with SZ may have more severe and widespread impairments.

## Introduction

In his landmark paper “The Diagnosis and Prognosis of Dementia Praecox” presented in Heidelberg in 1898 (1), German psychiatrist Emil Kraepelin discussed the results of his methodical observation of patients by stating that in dementia praecox “*The prognosis, however, is really by no means simple. Whether dementia praecox is susceptible of a complete and permanent recovery … is still very doubtful, if not impossible. But improvements are not at all unusual …. It is a more serious matter that in most of these cases the improvement is only temporary, and that such patients are in great danger of relapsing sooner or later, without any particular cause, and then generally suffer more serious injury from their illness*.” He distinguished dementia praecox from an apparently less severe course illness that was reported in 13% of his patients. The idea that an illness with a course potentially severe as dementia praecox and a “spontaneous recovery” was difficult to accept in the nineteenth century Germany psychiatry. In addition Kraepelin saw Dementia Praecox as a “*disease process in the brain, involving the cortical neurones* [*and brought about] by an autointoxication … as a result of a disorder of metabolism*” (2).

Although the course of illness, as understood by Kraepelin, pointed to a differentiate Manic Depressive Insanity (current bipolar disorder – BD) from Dementia Praecox (current Schizophrenia – SZ) in terms of degree of cognitive deterioration (Dementia), research in the last decade has begun to shed some light into the long term course of BD. In addition, they have also reported that in BD different cognitive domains deteriorate in remarkable similar ways as SZ. In other words, although Kraepelin’s great insight has led to the proper qualitative differentiation between these remarkably similar illnesses, recent prospective observational studies (3) have shown that BD and SZ share similar dimensional characteristics in terms of neurocognitive domains. Data from recent meta-analysis conducted in BD and SZ are in line with these findings. Depp et al. and Fett et al. reported a similar mean correlation between overall neurocognitive ability and everyday functioning (4, 5). Mann-Wrobel et al., similarly though less severely than in SZ (6), reported a broadly generalized cognitive impairment in euthymic BD patients with verbal ability as a possible area of preserved performance (7). Neuropsychological deficits represent a core feature of BD and SZ; however, their onset and progression differ between diagnostic groups. (8). For instance, although cognitive deterioration can appear from the outset in both disorders, in BD, as compared to SZ, it follows a progressive longitudinal worsening related to the number and the intensity of mood episodes (9). This might reflect different brain structural and functional alterations related to BD exacerbations. Imaging studies have shown alterations in regions related to emotional and cognitive functioning. A meta-analysis by Bora et al showed gray matter reductions are more circumscribed to these areas when controlling by gender. Modest differences between both disorders were found, with SZ patients showing higher dorsomedial and dorsolateral prefrontal cortex affectation compared with BD (10). In functional studies frontal-subcortical structures show abnormalities in both disorders with common hyper-activation of emotional limbic regions in relation to prefrontal-cortical regions. In addition, BD has shown more pronounced chronic hypo-activation of ventral-medial regions (11). The end result of this apparent connectivity abnormality may result in a glutamatergic transmission excess in key memory and processing regions with concomitant excitotoxicity and neurocognitive sequelae in BD (12). Moreover, modern techniques in molecular biology and neuroanatomical immunocytochemistry have established that both disorders share neuronal pathological markers in brain areas as hippocampus that correlate with neurocognitive sequelae (13, 14).

The aim of this systematic review is twofold: (i) describe and compare the neuropsychological dysfunction between BD and SZ patients as addressed by included studies; (ii) investigate the course of illness to better differentiate and predict onset and progression of these two disorders.

## Methods

To identify studies reporting cognitive impairment in BD and SZ we searched PubMed database for the period between 2008/01/31 and 2013/01/31, following PRISMA guideline recommendations (15). We combined the search strategy of free text terms and exploded MESH headings as following: [((SZ AND bipolar AND control) AND (neurocogniti^∗^ OR cognitive OR neuropsycholog^∗^)) NOT (Gen^∗^(MeSH Major Topic) OR Neuroimag^∗^(MeSH Major Topic))]. Additional studies were identified by reviewing the references of each article found, method deemed appropriated by recent articles seeking similar objectives as ours (4, 10). The search cutoff date was the last day of January 2013. Criteria for inclusion in this study were:(a) publication Full text available in English, (b) inclusion of a BD group with operationally defined euthymia, a schizophrenic group in remission and a healthy comparison group (studies that did not directly compare the three groups were excluded), (c) recruitment of adult population (18 years or older), (d) meta-analysis, controlled clinical trial, systematic reviews, comparative study, and randomized controlled trial, (e) assessment of the following cognitive functions: executive functions, memory, IQ or educational level, attention-concentration, and perceptuomotor function. Key words and application of inclusion/exclusion criteria resulted in 18 papers. Additionally we performed a second search using the algorithm (“premorbid” OR “pre-morbid” OR “at risk” OR “prodrom”) AND (schizoph^∗^ AND bipolar) AND (cognition OR neurocogniti^∗^ OR neuropsycholog^∗^) AND [Meta-Analysis(ptyp) OR Randomized Controlled Trial(ptyp) OR Review(ptyp) AND “last 5 years” (PDat)]. This algorithm yielded to one additional review (8) with same aims as ours. Additional 22 records were identified trough bibliographic cross-referencing. After removing 1 duplicate study, 39 records were screened. Application of inclusion/exclusion criteria resulted in exclusion of 29 studies: 16 were out the search interval dates, 2 lacked either schizophrenic or euthymic bipolar group, and 1 lacked of a control group, 3 were focused on differential neuroimaging. 2 were focused on genetics, and 4 on the effect of treatment alternatives (CBT, Befriending, ECT, and Ziprasidone), 1 studied only bipolar patients and discussed comparatively with SZ. As shown in Table 1, key words and application of inclusion/exclusion criteria finally resulted in 10 papers.

**Table 1:** **Included studies**.

Study	Sample characteristics	Materials
Barrett et al. (17) in Lewandowski et al. (8)	SZ (SZ = 44, SZA = 2, age = 29) BD/mania (*n* = 32, age = 37) HC (*n* = 67, mean age = 32)	Global intellectual functioning: WASI two-test version, vocabulary, and matrix reasoning Verbal attention and working memory (digit span forward/backward from WMS) Spatial attention: Corsi Block-Tapping Test Verbal memory: paired associates WMS Visual memory: ROCFT Response inhibition and attentional set shifting: Hayling and Brixton Tests Verbal fluency: COWATT Language: Perin’s Spoonerisms Callosal function: TFL
Brissos et al. (16)	SZ remitted (*n* = 23, mean age = 37) BD type I euthymic (*n* = 30, mean age = 36) HC (*n* = 23, mean age = 37)	Attention and mental control: WMS digit span Perceptual-motor skills: SDMT and TMT-A Executive functions: SCT and SCWT, TMT-B, and Verbal fluency: COWA Verbal abstraction: WAIS: comprehension and similarities subtests Visuo-spatial attention: Bell’s Test Memory function: WAIS: information subtest, WMS: logical memory
Brissos et al. (20)	SZ remitted (*n* = 35, mean age = 40) BD type I euthymic (HPS+ = 44, mean age = 39; HPS− = 35, mean age 38) HC (*n* = 50, mean age = 34)	Attention and mental control: mental tracking, digit span forward (WMS), Bell’s Test Processing speed: Symbol Digit Modalities Test, TMT-A Executive functions: Digit Span backward, SCT and SCWT, TMT-B, ToH, COWAT – animal naming Test of verbal fluency: WAIS-R comprehension and similarities subtests Verbal memory: WAIS-R – information subtest, WMS – logical memory
Gogos et al. (24)	SZ non-psychotic (*n* = 38, mean age = 43) BD euthymic (*n* = 40, mean age = 42) HC (*n* = 43, mean age = 41)	Immediate memory/learning, visuo-spatial ability, language, attention, and delayed memory: RBANS domain scores and total score
Joshua et al. (18)	SZ not acute phase (*n* = 39, mean age = 42) BD not acute phase (*n* = 40, mean age = 42) HC (*n* = 44, mean age = 41)	Executive functioning (response initiation, response suppression, and an error pattern): HSCT
Rossell and Batty (25)	SZ (*n* = 32, mean age = 36) BD/mania (*n* = 28, mean age = 39) HC (*n* = 32, mean age = 37)	Word comprehension across grammatical categories, i.e., nouns, verbs, and adjectives
Sanchez-Morla et al. (23)	BD (*n* = 73, mean age = 44) (BD I = 55, BD II = 18) SZ (*n* = 88, mean age = 39) HC (*n* = 67, mean age = 44)	Executive function. Concept formation and shifting: WCST. Planning and problem solving: ToH. Verbal fluency: COWAT. Semantic fluency: Animal Naming Test Executive control: TMT-B and SCWT. Verbal working memory: WAIS-R digit subtest, digits backward. Sustained attention. Degraded stimulus CPT Verbal learning and memory: CVLT Visual memory: ROCFT
Simonsen et al. (19)	SZ (*n* = 102, mean age = 32) SZA (*n* = 27, mean age = 34) BD I or II (*n* = 136, mean age = 36) BD with psychosis = 75 HC (*n* = 280, mean age = 36)	Verbal learning and memory: Logical Memory Test (WMS-III) and CVLT-II, with sub score measures of verbal learning and recall Processing speed: Digit Symbol Test WAIS-III Working memory: Digit Span Test – backward (WAIS-III) and WM-MA Verbal fluency: Verbal Fluency Test (D-KEFS), with measures of phonetic fluency, semantic fluency, and semantic fluency set shift Interference: Color Word Interference Test (D-KEFS), with subscore for interference control and interference set shift
Wobrock et al. (22)	SZ first episode (*n* = 11, mean age = 30) SZ chronic (*n* = 13, mean age = 35) BD (*n* = 18, mean age = 42) HC (*n* = 23, mean age = 30)	Deficits in attention, speed processing, and psychomotor performance: RT1 single stimulus and RT3 compound stimulus (subtests of the WTS) Divided attention task: “Geteilte Aufmerksamkeit” (subtest of TAP) Psychomotor performance and speed: ZVT, a test similar to the TMT-A Attention and working memory: digit span task “Zahlennachsprechen” (a subtest of the HAWIE), working memory test “Arbeitsgedächtnis” (subtest of the TAP) Visuo-spatial working memory: Corsi Block-Tapping Test (of WTS battery), Test of Spatial Recognition Memory (CANTAB) Verbal learning and memory: VLMT, similar to the CVLT Non-verbal learning capacity: NVLT, subtest of WTS Verbal fluency: RWT Deficits in concept formation, cognitive flexibility, and executive functions: WCST and TOL Intermodal comparison: “Intermodaler Vergleich” (subtest of the TAP). Interference: “Stroop” task (subtest of the WTS), word (Stroop-LI) and color (Stroop-BI) interference conditions
Zanelli et al. (28)	SZ (*n* = 65, mean age = 27) BD (*n* = 37, mean age = 28) OP (*n* = 46, mean age = 29) Depressive psychosis (*n* = 39, mean age = 37) HC (*n* = 177, mean age = 37)	Learning and memory: RAVLT, visual reproduction subtest of the WMS–R Executive functions and working memory: TMT-B; LNST, RCPM series AB and B Attention, concentration, and processing speed: TMT-A and WAIS-R digit symbol subtest Language: WAIS-R category fluency (semantic and letter fluency tasks) Visual-spatial perception and organization: set A of RCPM and WAIS-R Block Design Verbal intelligence: WAIS-R vocabulary and comprehension subtests

*HC, healthy controls; SZ, schizophrenia; BD, bipolar disorder; SZA, schizoaffective disorder; SZF, schizophreniform disorder; HPS+, with history of psychosis; HPS−, without history of psychosis; OP, other psychotic disorders (persistent delusional disorders, acute and transient psychotic disorders, other non-organic psychotic disorders, and unspecified non-organic psychosis)*.

CANTAB, Cambridge Neuropsychological Test Automated Battery; COWAT, Controlled Oral Word Association Test; CPT, Continuous Performance Test; CVLT, California Verbal Learning Test; D-KEFS, Delis–Kaplan Executive Function System; DP, Depressive Psychosis; HSCT, Hayling Sentence Completion Test; LNST, Letter-Number Span Test; NVLT, “Non-verbaler Lerntest”; RAVLT, Rey Auditory Verbal Learning Test; RBANS, Repeatable Battery for the Assessment of Neuropsychological Status; RCPM, Raven’s Colored Progressive Matrices; ROCFT, Rey–Osterrieth Complex Figure Test; RT1, “Reaktionstest” single stimulus; RT3, “Z” compound stimulus; RWT, “Regensburger Wortflüssigkeits-Test”; SCT, Stroop Color Test; SCWT, Stroop Color-Write Test; TFLT, Tactile Finger Localization Test; SDMT, Symbol Digit Modalities Test; TAP, “Aufmerksamkeitsprüfung” test battery; TMT-A, Trail Making Test part A; TMT-B, Trail Making Test part B; ToH, Hanoi Towers Test; TOL, Tower of London Test; VLMT, “Verbaler Lern und Merkfähigkeitstest”; Voc, WAIS Vocabulary subtest; WAIS-R/-III, Wechsler Adult Intelligence Scale (Revised/Third Edition); WASI, Wechsler Abbreviated Scale of Intelligence; WCST, Wisconsin Card Sorting Test; WM, working memory; WM-MA, Working Memory-Mental Arithmetic Test; WMS, Wechsler Memory Scale; WTS, Wiener Test System; ZVT, “Zahlenverbindungstest.”

Two reviewers extracted all data independently (Sergio A. Barroilhet and Paul A. Vöhringer). Discrepancies between reviewers were resolved through discussion until consensus was reached.

## Results

Findings from studies that analyzed the cognitive profile of patients with SZ and BD when compared with healthy controls (HC) are summarized in Table 2.

**Table 2:** **Findings**.

Study	Executive functions and language	Memory	Attention	Perceptuomotor functions	IQ	Notes
Barrett et al. (17) in Lewandowski et al. (8)	BD, SZ < HC, impaired on executive functioning and language by first episode SZ < BD < HC on response inhibition, verbal fluency and callosal functioning	BD, SZ < HC, impaired in verbal and visual memory performances by first episode BD pt are reported to have intermediate results between the two groups	No differences		BD and SZ pt disclosed lower premorbid and current IQ scores compared to HC BD > SZ in current IQ score	SZ with “preserved” IQ = BD: similar “pattern” of deficits
Brissos et al. (16)	BD, SZ < HC in stimulus inhibition, and information processing BD pt performed intermediately between SZ pt and HC although differences didn’t reach statistical significance	No differences	SZ < BD, HC on simple automatic and attentional processing Attention deficits in SZ and BD pt persisted during the entire course of the disease including periods of euthymia	BD, SZ < HC on perceptual-motor skills	Not assessed	Although not significantly, BD pt performed intermediately between pt with SZ and HC
Brissos et al. (20)	SZ < BD < HC on mental control, processing speed, executive functions (only quantitative, not qualitative differences)	SZ, BD < HC on verbal memory function	SZ > BD HPS+, HPS−, HC in slowness and perseveration in incongruent tasks is interpreted as a proxy of deficient inhibitory control, strongly related with poor selective attention	Not assessed	Controlled for educational level, not IQ	BD HPS+ was not associated with more severe cognitive impairment during euthymia
Gogos et al. (24)	SZ, BD < HC on language	SZ < BD < HC on immediate memory/learning SZ < BD, HC on delayed memory SZ < BD, HC on visuo-spatial and delayed memory	SZ < HC on attention SZ = BD on attention Attention deficits in SZ and BD patients persisted during the entire course of the disease including periods of euthymia. Similar results found using different tests that required keeping attention focused for an extended amount of time	Not assessed	Matched for IQ	SZ < BD, HC on overall cognitive functioning
Joshua et al. (18)	SZ < BD, control on overall HSCT performance (overall executive dysfunction) SZ > HC on response latency (BD scored in the middle with ns. differences with either SZ or control) SZ > HC on response errors (BD scored in the middle with ns. differences with either SZ or control) SZ > BD, HC on semantically related errors	Not assessed	Not assessed	Not assessed	Controlled for IQ	SZ group was significantly impaired on all measures of executive functioning BD group did not differ, from HC
Rossell and Batty (25)	SZ < BD, HC on the semantic retrieval (more executive task) SZ = BD = HC on word comprehension across grammatical categories (depend on executive regulation)	SZ, BD < HC on semantic recognition, both clinical groups presented similar performance with difficulties in identifying the correct word meaning in the presence of alternatives	Not assessed	Not assessed	BD and HC matched for IQ, and different from SZ Controlled for IQ	SZ and BD show a shortfall in ability to organize and categorize word meanings
Sanchez-Morla et al. (23)	SZ, BD < HC on all tasks SZ < BD: executive control, verbal working memory SZ = BD on executive control, planning and problem solving, and semantic fluency	SZ < BD < HC in immediate and delayed visual memory SZ vs. BD: mixed results in Verbal Memory Tests	SZ, BD < HC Attention deficits in SZ and BD pt persisted during the entire course of the disease including during periods of euthymia	Not assessed	SZ, BD < HC Controlled for IQ	Duration of illness correlated with executive, verbal memory and visual memory functioning in BD BD with history of psychosis = BD without history psychosis
Simonsen et al. (19)	SZ, SZA, BD HPS+,<BD, HPS−, HC on set shifting verbal fluency BD HPS− = HC on all neurocognitive subscores, apart from digit symbol where they performed poorer	SZ, SZA, BD with psychosis<BD, HC on verbal learning and memory BD pt are reported to have intermediate results between the two groups	Not assessed	Both psychotic and non-psychotic pt reported lower perceptuomotor functions as compared to HC. No significant differences were shown between them. Other pt with a history of psychosis (SZ, SZA) performed poorly than HC on measures of processing speeding. In particular, SZ pt showed lower Dygit Symbol Test – WAIS-III scores as compared to psychotic BD pt	SZ < BD, HC Controlled for IQ	All patients with a history of psychosis performed poorly on all measures
Wobrock et al. (22)	SZ chronic and first episode<HC on speed, verbal fluency, and verbal working memory With regard to cognitive flexibility, SZ pt showed more perseverative responses with poorer WCST scores as compared to BD pt and HC	Both first episode and chronic SZ pt have decreased verbal learning and memory performances as compared to HC. BD pt are reported to have intermediate results between the two groups	SZ pt chronic and first episode<HC on attention	SZ pt chronic and first episode<HC on psychomotor performance	SZ pt showed significant poorer results compared to HC	SZ pt overall showed a poorer performance than BD and HC
Zanelli et al. (28)	SZ < BD on measures of vocabulary and comprehension, digit symbol, letter-number span, and block design BD < HC on measures of category fluency SZ < HC on the Letter-Number Span Test DP < HC on measures of verbal learning and category fluency, and on the TMT-B OP < HC on verbal learning BD < HC on category fluency	BD < HC on measures of delayed verbal memory	SZ < BD < HC in overall measures	Not assessed	SZ pt showed significant poorer results compared to HC in all examined studies Similar pattern between clinical groups were reported with regard to current IQ	SZ acute and chronic showed poorer performance than the rest

*HC, healthy controls; SZ, schizophrenia; BD, bipolar disorder; SZA, schizoaffective disorder; SZF, schizophreniform disorder; Pt, patients; NART, National Adult Reading Test; SDMT, Symbol Digit Modalities Test; TMT-A, Trail Making Test part A; TMT-B, Trail Making Test part B; HDRS, Hamilton Depressive Rating Scale; YMRS, Young Mania Rating Scale; SCWT, Stroop Color-Write Test; CPT, Continuous Performance Test; WAIS-R, Wechsler Adult Intelligence Scale Revised; WCST, Wisconsin Card Sorting Test*.

Reviewed studies found that overall both SZ and BD patients present deficits on almost all neurocognitive measures compared to HC, with few exceptions in some domains (16, 17) for some sample comparisons (18, 19). In particular, SZ patients show more severe and pervasive cognitive deficits while BD patients present a milder and more confined impairment (20). In addition, evidence from the literature suggests that SZ and BD patients share a similar cognitive impairment profile with different degrees of deficits. Therefore, the difference between the two groups seems to be more quantitative (degree of deficit) rather than qualitative (profile), supporting a dimensional approach to the two clinical entities (21).

With regard to the course of the disease, authors report that in BD patient’s cognitive deficits persist during periods of euthymia, which implies that cognitive deficits are not uniquely associated with the clinical manifestations of BD. In this context, neurocognitive impairment would be considered a trait rather than a state-specific feature.

### Executive functions

Cognitive flexibility, inhibitory control, working memory and fluency, among others, were considered.

To analyze executive functions authors used tests that assess cognitive flexibility through attention shifting tasks (Trail Making Test – Part B; TMT-B, Brixton Spatial Anticipation Test; BSAT, Wisconsin Card Sorting Test; WCST) and tests that assess inhibitory control through selective attention tasks (Stroop Color Test; SCT, Stroop Color-Write Test; SCWT, Hayling Sentence Completion Test; HSCT).

With regard to cognitive flexibility, SZ patients showed more perseverative responses with poorer WCST scores as compared to BD patients and HC after adjusting for gender, age, and duration of education (*p* = 0.002 and 0.026, respectively) (22).

Brissos et al., Joshua et al., Sanchez-Morla et al., reported that BD patients performed intermediately between SZ patients and HC on stimulus inhibition and speed information processing (16, 18, 23).

Studies investigating working memory used a variety of different tests (Attention Test Battery; TAP, Digit Spam Test; WAIS-III, Working Memory-Mental Arithmetic Test; WM-MA). They showed that SZ patients performed poorly as compared to HC (19, 22). In addition, Sanchez-Morla et al. emphasize significant differences between SZ and BD patients with worse results in the former group (Digits Backward – WAIS-R: 4.5 (±1.9) and 4.9 (±1.9), respectively, *p* < 0.003; after adjusting for age, years of education, gender, subclinical depressive symptomatology, and premorbid IQ) (23).

Similar trends were reported when analyzing verbal fluency; SZ patients performing worse relative to HC (Verbal Fluency Test, D-KEFS: phonetic: 37.8 (±12.3) vs. 44.9 (±10.8), semantic: 39.0 (±10.6) vs. 48.1 (±8.4), set shifting: 12.0 (±2.5) vs. 14.7 (±2.7), *p* < 0.001, after adjusting for demographic variables, current symptom measures, and illness course variables) (19). BD patients performed intermediately between the two groups (17).

### Memory

There is strong evidence that both first episode and chronic SZ patients have decreased verbal learning and memory performances as compared to HC. BD patients are reported to have intermediate results between the two groups (17, 19, 22–25).

In particular, a study conducted in Australia on 32 SZ and 28 BD patients explored the semantic retrieval (Generate condition) and the semantic recognition (Forced choice condition): SZ patients performed significantly poorer compared to HC on semantic retrieval (MRC Oxford Psycholinguistic Database Generate condition total score: 49.7 (±20.4) and 74.1 (±17.8), respectively, *p* < 0.001; after adjusting for premorbid IQ) (25). Similar findings were reported in other studies (26, 27). On the contrary, BD patients did not present significant impairment. With regard to semantic recognition, both clinical groups presented similar performance with difficulties in identifying the correct word meaning in the presence of alternatives (MRC Oxford Psycholinguistic Database Forced choice condition total score: 72.7 (±16.0) and 75.1 (±17.0), respectively vs. 88.0 (±5.6) in HC, *p* < 0.001; after adjusting for premorbid IQ) (25).

Sanchez-Morla et al. examined visual memory on 88 SZ and 73 BD patients using Rey-Osterreith Complex Figure Test (ROCFT). They found significant differences in immediate and delayed visual memory among the three groups, with BD patients performing intermediately between SZ patients and HC (23).

### IQ

Several studies analyzed premorbid IQ in SZ and BD patients using tests based on measures of current ability though to be relatively insensitive to brain dysfunction (17, 23).

Patients affected by SZ showed significant poorer results compared to HC in all examined studies (22, 28). Two studies showed that BD patients disclosed lower scores compared to HC (Vocabulary Subtest – WAIS-R: 103.8 (±19.5) and 108.9 (±17.8), respectively, *p* < 0.0001; National Adult Reading Test – NART IQ: 103.29 (±11.88) and 107.74 (± 9.98), respectively, *p* < 0.05) (17, 23). Similar pattern between clinical groups were reported with regard to current IQ (17, 28).

### Attention

Some authors reported that attention deficits in SZ and BD patients persisted during the entire course of the disease including during periods of euthymia (16, 23, 24, 29, 30).

Researchers described similar degree of sustained attention impairment in SZ and BD patients. In particular, a study conducted in Spain in 2009 measured – using the Continuous Performance Test (CPT) – lower number of hits and a longer reaction time in both SZ and BD patients as compared to HC (CPT: hits: 26.2 (±10.4), 30.7 (±8.7), and 34.6 (±6.2), respectively, *p* < 0.037; Furthermore, the reaction time was longer for SZ and BD compared with HC (ms): 550 (±94), 540 (±98), and 480 (±65), respectively, *p* < 0.0001; after adjusting for the effect of age, years of education, gender, subclinical depressive symptomatology, and premorbid IQ) (23). Others reported similar finding using different tests that required keeping attention focused for an extended amount of time (Repeatable Battery for the Assessment of Neuropsychological Status; RBANS, Subtest Attention, Digit Spam, and Coding) (24).

Based on results of the Stroop Test, Brissos et al. described increased slowness and perseveration in incongruent tasks in SZ patients as compared to BD patients (with or without history of psychotic symptoms) and HC and interpreted it as a proxy of deficient inhibitory control, strongly related with poor selective attention (20).

### Perceptuomotor functions

Simonsen et al. divided the BD study population in psychotic and non-psychotic subjects. Both categories reported lower perceptuomotor functions as compared to controls. No significant differences were shown between them. Other patients with a history of psychosis (SZ, schizoaffective disorder) performed poorly than HC on measures of processing speeding. In particular, SZ patients showed lower Dygit Symbol Test – WAIS-III scores as compared to psychotic BD patients [56.1 (±14.5) and 63.7 (±16.2), respectively, *p* < 0.001; after adjusting for demographic variables, current symptom measures, illness course variables]. These results are consistent with findings from Brissos et al. that, using different tests, revealed a significant worse performance for SZ patients relative to HC [Symbol Digit Modalities Test – SDMT: 30.6 (±11.71) and 56.4 (±14.82), respectively, *p* < 0.001; Trail Making Test part A – TMT-A: 78.8 (±50.76) and 38.0 (±15.34), respectively, *p* < 0.001; after adjusting for sex, age, educational level, Hamilton Depressive Rating Scale – HDRS, Young Mania Rating Scale – YMRS, as covariates) (20). Evidence from Germany supported such patterns reporting lower performances for both first-episode and chronic SZ patients as compared to HC (22).

## Discussion

Overall, patients with BD exhibit neurocognitive impairment qualitatively similar to but quantitatively less pronounced than patients with SZ. In a general sense, it seems that while emotional and interpersonal difficulties in childhood reflect a general predisposition to adult psychiatric illness, early developmental impairments in psychomotor, language, and cognitive function show some specificity to SZ outcomes and are not seen in those who later develop BD. (31)

A previous meta-analysis showed that SZ patients demonstrate worse cognitive performance than BD patients in most domains, with substantial heterogeneity. This study focused on performance differences between patients groups, without normal controls, thus deviations from the norm were not analyzed (32). The course of cognitive impairment also differs between groups: premorbid cognitive impairment is present early in life in SZ (often childhood/adolescence), preceding onset of psychosis, but cognitive impairment typically does not occur until later in adulthood in BD, after many years of mood episodes (8).

Despite overlap between BD and SZ in genetics and phenomenology of psychosis (31), these cognitive impairment data indicate that BD and SZ are different in severity and course of cognition.

During adulthood, BD patients have shown impairment on neuropsychological measures sensitive to frontal lobe dysfunction during the adolescence, usually before the diagnosis is made. This may suggest the presence of some kind of neurodevelopmental vulnerability, expressed initially as attentional deficits or comorbidity with attention deficit-hyperactivity disorder (33). Nevertheless, there is lack of systematic studies regarding time varying patterns upon developmental deficits, starting on pre-morbid stage, and continuing during the illness phases of both conditions. Regarding specific domains of cognitive impairment, in SZ working memory was impaired, while in BD attention was impaired (34)

The usual clinical symptomatology showed by these illnesses can also modify cognitive functioning. For instance, severity of negative symptoms in SZ may have some correlation with poor neurocognitive performance, while on the contrary, severity of depressive symptoms in BD showed no association with performances on any neuropsychological test (28). A high level of negative symptomatology might be seen as predictor of worse response (17, 19); there are prior data suggesting that negative symptoms had strong predictive capacity of neurocognitive outcomes associated with measures of executive functioning and selective attention, but not associated with visual or verbal learning and memory, verbal fluency, or processing speed (35). Further, changes in depressive symptoms were correlated with poorer verbal fluency, while no relationship between manic symptoms and neuropsychological performance was detected (36).

Evidence suggests that patients were usually taking psychiatric medication while cognitively assessed, eventually biasing the measure of differences between groups. This lack of clean assessment, impede drawing any definitive conclusion of the effects of medication on cognitive function. One study suggested the presence of the pharmacological treatment over cognitive functioning. (37) concluded that schizophrenic patients in polypharmacy had significantly lower cognitive functions than those in the second-generation antipsychotics monotherapy (37). Additionally, moderate improvement was found in cognitive test performance of patients on their first episode of SZ treated with second-generation antipsychotics (38). Similarly, untreated bipolar patients showed better cognitive performance than patients on atypical antipsychotics (39).

Interesting evidence on the effect of psychopharmacologic agents over cognitive function has been developed with lithium. Studies suggest that cognitive deficits are not explained by this medication, but by the condition itself (15, 40). With respect to other drugs, cognitive side effects may occur as a direct consequence of the mechanism of action of the psychotropic agent, or indirectly through side effects such as sedation (41). Nevertheless, post-onset neuropsychological evaluation may be confounded by medication, making it difficult to comment about the relevance of neuropsychological deficits when talking about the etiology or development of these conditions. We think that further randomized research on drug naive patients should clarify the role of medication on cognitive deficits. Moreover, typically early models for the etiology of psychotic illnesses focused on dopamine neurotransmission because of the powerful antipsychotic action of dopamine antagonists giving support to the so-called dopamine hypothesis of psychosis. Nevertheless, recent evidence increasingly supports a primarily glutamatergic dysfunction in this condition, where dopaminergic disbalance is a secondary effect. (42) In fact, a more recent model for the pathophysiology of SZ and BD involves a pathophysiologic mechanism by which the cognitive decline is representing the core of the clinical morbidity and in this context impairment of glutamate and more specifically *N*-methyl-d-aspartate (NMDA) function has been considered of major importance. Given that astrocytes are crucial in controlling glutamate homeostasis, seems to logical to assign a major role to glial–neuronal interactions in the pathophysiology of the cognitive decline in these conditions (43)

Few studies evaluated psychosocial function of patients. When assessed, verbal memory impairment seemed to correlate with a poorer psychosocial functionality. Furthermore, BD patients scored lower than SZ patients; we thought that depressive or sub threshold depressive symptoms might play a role on these results. Executive function had a directly positive effect on psychosocial adjustment in both patients with BD and SZ. There were positive correlations between insight and executive function among subjects with SZ and BD, suggesting that the ability to recognize their own experiences of illness seems to be the first step to developing an effective strategy for supplementing their deficiencies in psychosocial adjustment (44). The possibility of a shared general cognitive impairment might yet be possible as an explanation, due to comparable qualitative scores shown by the two conditions; nevertheless these abnormalities oftentimes are larger in SZ patients. Following the same vein, a study suggests the existence of a continuum between SZ and affective disorders but with substantial difference between the courses of their cognitive deficits, being more severe in SZ and milder in BD (28). On the other hand, a study contradicts this theory suggesting that BD patients usually present impaired performance on multiple cognitive domains, whereas their first-degree relatives shown performances comparable to HC on most cognitive tasks (3) additional research is warranted in order to reach a sound conclusion on this matter.

Evidence regarding the effect of past psychotics symptoms on the neurocognitive performance remains unclear. A study found similar performance in all BD patients; no differences were found depending on the history of the disease (16). On the contrary, there is evidence concluding that patients with a psychotic bipolar condition represent a group that is neurocognitive closer to the SZ than the non-psychotic bipolar subjects. This study was carried out with symptomatic patients and therefore this clinical condition may confound these results (19).

The overall homogeneity among these findings argues favorably for the validity of a milder neuropsychological impairment in BD compared with SZ and yet other considerations like the eventual cognitive continuum between BD and SZ, the role of psychotropic medication, the pre-morbid deficit and the possible effect of psychotic symptomatology over the neuropsychological status remains unclear.

Limitations of the present systematic review includes heterogeneity of samples and assessments, the impossibility to control for acute or chronic effects of medication, varying time required for assessment across different studies, illness diagnosis stability, and course severity. Another limitation to be considered pertains with the time limit that was set up for the systematic review. In effect, to gain novelty about the selected studies, search time was set up with year 2008 as the lower limit and important papers about glial research have appeared before the 2008, thus some relevant evidence regarding this pathophysiological mechanism may be left out of the overall search.

## Conflict of Interest Statement

S. Nassir Ghaemi has received a research grant from Pfizer. Neither he nor his family holds equity positions in pharmaceutical corporations. The other authors declare that the research was conducted in the absence of any commercial or financial relationships that could be construed as a potential conflict of interest.
